# Evogliptin Suppresses Calcific Aortic Valve Disease by Attenuating Inflammation, Fibrosis, and Calcification

**DOI:** 10.3390/cells10010057

**Published:** 2021-01-01

**Authors:** Bongkun Choi, Eun-Young Kim, Ji-Eun Kim, Soyoon Oh, Si-On Park, Sang-Min Kim, Hyuksu Choi, Jae-Kwan Song, Eun-Ju Chang

**Affiliations:** 1 Department of Biomedical Sciences, Asan Medical Center, University of Ulsan College of Medicine, Seoul 05505, Korea; bkchoi89@hanmail.net (B.C.); kimberly_kim44@hotmail.com (E.-Y.K.); kge33@sookmyung.ac.kr (J.-E.K.); ohwlsgml@naver.com (S.O.); sionirena@naver.com (S.-O.P.); rlatkals90@gmail.com (S.-M.K.); nga1995@naver.com (H.C.); 2 Stem Cell Immunomodulation Research Center, Asan Medical Center, University of Ulsan College of Medicine, Seoul 05505, Korea; 3 Division of Cardiology, Asan Medical Center, University of Ulsan College of Medicine, Seoul 05505, Korea; jksong@amc.seoul.kr; 4 Department of Biochemistry, Asan Medical Center, University of Ulsan College of Medicine, Seoul 05505, Korea

**Keywords:** calcific aortic valve disease, dipeptidyl peptidase-4, aortic valve, inflammation, fibrosis, calcification

## Abstract

Calcific aortic valve disease (CAVD) accompanies inflammatory cell infiltration, fibrosis, and ultimately calcification of the valve leaflets. We previously demonstrated that dipeptidyl peptidase-4 (DPP-4) is responsible for the progression of aortic valvular calcification in CAVD animal models. As evogliptin, one of the DPP-4 inhibitors displays high specific accumulation in cardiac tissue, we here evaluated its therapeutic potency for attenuating valvular calcification in CAVD animal models. Evogliptin administration markedly reduced calcific deposition accompanied by a reduction in proinflammatory cytokine expression in endothelial nitric oxide synthase-deficient mice in vivo, and significantly ameliorated the mineralization of the primary human valvular interstitial cells (VICs), with a reduction in the mRNA expression of bone-associated and fibrosis-related genes in vitro. In addition, evogliptin ameliorated the rate of change in the transaortic peak velocity and mean pressure gradients in our rabbit model as assessed by echocardiography. Importantly, evogliptin administration in a rabbit model was found to suppress the effects of a high-cholesterol diet and of vitamin D2-driven fibrosis in association with a reduction in macrophage infiltration and calcific deposition in aortic valves. These results have indicated that evogliptin prohibits inflammatory cytokine expression, fibrosis, and calcification in a CAVD animal model, suggesting its potential as a selective therapeutic agent for the inhibition of valvular calcification during CAVD progression.

## 1. Introduction

Calcific aortic valve disease (CAVD) is a progressive disorder that ranges from the fibrous thickening of valve leaflets to severe calcification accompanied by impaired leaflet motion or aortic valve stenosis [1]. CAVD is one of the most common valvular heart disorders, but no medical therapies that can halt or delay its progression are currently available, other than aortic valve replacement or implantation [2,3]. Clinicopathological studies suggest that initiation and progression of CAVD can be seen as distinct phases: endothelial dysfunction and lipid deposition, inflammation, fibrosis, osteogenic differentiation, and end-stage calcification. In the first initiation phases, there is lipid deposition accompanied by inflammation. In the second progression phase, thickening and stiffening of the valve leaflets attributable to fibrosis, osteogenic differentiation of valvular interstitial cells (VICs), and calcification on the surface of the leaflet are responsible for disease progression [4,5,6]. Myocardial fibrosis also facilitates progressive systolic and diastolic impairment leading to heart failure [7].

Dipeptidyl peptidase-4 (DPP-4) cleaves two amino acids from the N-terminus of its target substrates that have an alanine or proline in the second position [8,9]. Because the major targets of DPP-4 are incretin hormones that regulate insulin secretion, DPP-4-inhibitors have been broadly used to treat type 2 diabetes mellitus [10,11]. Nevertheless, DPP-4 has various biological functions and can proteolytically inactivate a number of other mediators in addition to incretin hormones. Accumulating evidence has now suggested that the beneficial effects of DPP-4 inhibitors include not only type 2 diabetes, but also specific cardiovascular diseases [12,13]. Indeed, DPP-4 inhibitors have been found to significantly suppress inflammatory cytokine gene expression [14], and several lines of evidence have indicated the anti-inflammatory properties of DPP-4 inhibitors [15,16,17]. In addition, DPP-4 inhibitors significantly suppress cardiac myocardial fibrosis [14,18].

We recently reported that endothelial dysfunction in the aortic valve increases DPP-4 expression, thereby leading to induced degradation of insulin-like growth factor-1 (IGF-1) and the subsequent osteogenic differentiation of VICs, and that sitagliptin, a selective DPP-4 inhibitor that has been broadly used for the treatment of human type 2 diabetes mellitus, hindered the progression of aortic valve calcification in CAVD animal models [19]. This implied that DPP-4 inhibitors may be useful for preventing or delaying CAVD progression. Various DPP-4 inhibitors are currently available and we showed that the distribution of evogliptin in cardiac tissue is considerably higher than the other seven DPP-4 inhibitors, in another report from our laboratory [20]. We thus speculated that evogliptin, a DPP-4 inhibitor possessing high cardiac tissue distribution profile, may be effective as a disease-modifying agent for CAVD.

Given our previous evaluation of the cardiac tissue distribution profile of eight different DPP-4 inhibitors, and finding that evogliptin had the highest adjusted heart to plasma concentration [20], we here studied the effects of evogliptin on aortic valve inflammation, fibrosis, and calcification in CAVD animal models. In our present analyses, we evaluated the efficacy of evogliptin in suppressing the in vitro osteogenesis of VICs, inflammatory cytokine expression, and the formation of calcific lesions in CAVD animal models to elucidate the mechanism by which DPP-4 inhibitor attenuates CAVD.

## 2. Materials and Methods

### 2.1. Human Subjects and Isolation and Culture of Human Valve Interstitial Cells

To isolate human valve interstitial cells (VICs), aortic valves tissues were first collected from patients who underwent aortic valve replacement at Asan Medical Center. Human VICs were then isolated from the aortic valve leaflets by enzyme isolation and maintained in Dulbecco’s Modified Eagle Medium (DMEM, Thermo Fisher Scientific, Waltham, MA, USA) containing 10% fetal bovine serum (FBS, Thermo Scientific) and penicillin/streptomycin (Life Technologies, Carlsbad, CA, USA) at 37 °C in a humidified 5% CO_2_ atmosphere, as previously described [21,22,23]. Cells at passages 3 to 10 were utilized in the analysis, and calcification of the VICs was induced as previously described [24]. All human subjects were treated in accordance with the Declaration of Helsinki and human samples were collected using a protocol approved by the Institutional Review Board (IRB) of Asan Medical Center (Seoul, Korea) (reference No. 2013-0442). All human subjects provided written informed consent to participate, including consent to publish.

### 2.2. Mice

All animal procedures were conducted in accordance with the protocols approved by the Institutional Committee for the Use and Care of Laboratory animals of Ulsan University (2018-12-074). We purchased endothelial nitric oxide synthase-deficient (*eNOS*^−/−^) mice in the C57BL/6 background from Jackson Laboratories (Bar Harbor, ME, USA) to utilize as an animal model of CAVD [25,26]. These mice were maintained on a 12 h light–dark cycle and were randomly assigned at 8 weeks of age to an evogliptin-treated (*n* = 6) and untreated (*n* = 6) group. Evogliptin dissolved in saline was orally administered at 1 mg/kg/day once daily for 12 weeks. The untreated group was orally administered with vehicle (saline). Evogliptin was provided by Dong-A ST research institute (Korea, Lot No. 51912001) with a material transfer agreement.

### 2.3. Rabbits

Nineteen male New Zealand white rabbits (weight, 2.5–3.0 kg) were divided into three groups in accordance with dietary intake and evogliptin administration and followed for 12 weeks: (1) rabbits were fed normal chow (control group, *n* = 5); (2) rabbits were fed a 0.5% cholesterol-enriched chow (Dyets Inc., Bethlehem, PA, USA) plus 25,000 IU/day vitamin D2 (VitD; Santa Cruz Biotechnology, Santa Cruz, CA, USA) (high fat diet (HFD) + VitD group, *n* = 7); and (3) rabbits were fed HFD + VitD and administered with evogliptin (1 mg/kg/day) orally for 12 weeks (evogliptin group, *n* = 7). Blood samples were obtained from the marginal vein of the ear. After 12 weeks, the rabbits were euthanized and both heart tissue and blood samples were obtained for analysis.

### 2.4. Osteogenic Differentiation

To induce the osteogenic differentiation of human VICs, osteogenic media (DMEM supplemented with 0.25 mM L-ascorbic acid, 10 mM β-glycerophosphate, and 10 nM dexamethasone) was applied for 21 days with fresh replacement every 3 days.

### 2.5. Alizarin Red Staining

Osteogenic differentiation and calcific deposition in the VICs was determined by alizarin red staining as previously described [27]. Briefly, cells were washed three times with PBS, fixed with 4% formaldehyde for 30 min, and incubated with 2% alizarin red staining solution (Sigma, St. Louis, MO, USA).

### 2.6. Calcium Assay

Calcium concentrations were determined colorimetrically utilizing the o-Cresolphthalein method with 0.1 M HCL extracts from VICs [28].

### 2.7. DPP-4 Activity Assay

DPP-4 activity in rabbit plasma was determined using a DPP-4 activity assay kit (Calbiochem, San Diego, CA, USA) in accordance with the manufacturer’s instructions.

### 2.8. Enzyme-Linked Immunosorbent Assay (ELISA)

The concentrations of Tumor necrosis factor-α (TNF-α, MTA00B), Interleukin-1β (IL-1β, MLB00C), and IL-6 (M6000B) proteins in mouse plasma were evaluated using sandwich ELISA systems (R and D), in accordance with the manufacturer’s protocols. All samples were examined in triplicate for each experiment.

### 2.9. Fluorescence Reflectance Imaging In Vivo

The experimental mice were administered a bisphosphonate-conjugated imaging agent (Osteosense680, VisEn Medical Inc., Bedford, MA, USA) or saline (control) via tail vein injections at 24 h prior to imaging to detect osteogenic activity [29,30]. Osteosense680 binds to calcification sites, particularly to hydroxyapatite, and thereby serves as an imaging agent for the detection of osteoblast function and calcific deposition in vivo [30,31]. After the animals had been euthanized, calcific deposits were imaged utilizing OptixMX3 (Advanced Research Technologies Inc., Saint-Laurent, QC, Canada).

### 2.10. Quantitative Real-Time Polymerase Chain Reaction (qPCR)

Total RNA was isolated from the VICs using a RNeasy kit (Qiagen, Germantown, MD, USA) following the manufacturer’s instructions. First-strand cDNA was then synthesized using the RevertAid First strand cDNA Synthesis kit (Thermo Scientific, EU) in a BIO-RAD T100TM-Thermal Cycler (Bio-Rad, Hercules, CA, USA). qPCR analysis was carried out using SYBR Green PCR master mix (Roche, Penzberg, Germany) in a Light Cycler 480 Real time-PCR Detection system (Roche), according to the manufacturer’s protocol. The results were presented as the ratio of target PCR products relative to glyceraldehyde-3-phosphate dehydrogenase (GAPDH), which was used as an internal control. The relative expression of the target genes was calculated utilizing the comparative threshold cycles (2^−ΔΔCt^) method.

### 2.11. Immunohistochemistry

Serial 5-μm paraffin embedded sections were prepared from rabbit tissues and immunohistochemical staining analysis with anti α-smooth muscle actin (α -SMA) antibody (Abcam, ab240654, Cambridge, MA, USA) or rabbit anti macrophage antibody (Dako, M0633, Santa Clara, CA, USA) was performed using the 5 REAL^TM^ EnVision^TM^ Detection System Peroxidase/DAB+ Detection System kit (Dako), following the manufacturer’s instructions. Hematoxylin and eosin (H/E) stains were then performed (Sigma, St. Louis, MO, USA) and Masson’s trichrome (MT, Sigma) was used to detect extracellular matrix deposition. Tissues were photographed with an Olympus BX51 microscope (Olympus, Center Valley, PA, USA).

### 2.12. Echocardiography

Echocardiography was performed in CAVD rabbits that had been anesthetized using an intramuscular administration of ketamine (30 mg/kg) and xylazine (6 mg/kg). The transvalvular gradient and aortic valve area were determined as described previously [32]. Briefly, the aortic valve area was determined using the standard continuity equation. The diameter of the left ventricular outflow tract was determined in the parasternal long axis view. The stroke volume with a pulsed-Doppler proximal to the aortic valve was measured in an apical five-chamber view. In addition, a continuous-wave Doppler was obtained to measure the integral of the transvalvular flow to be used in the continuity equation for aortic valve area calculation, as well as to record the maximal and mean trans-aortic pressure gradients.

### 2.13. Statistical Analysis

All quantitative experiments were performed at least three times. The study results were expressed as a mean ± SEM or mean ± SD. In vitro data were normally distributed, with similar variances among the groups. Due to the small number of animals in each group, and because some of the distributions were not normal in the animal study, a nonparametric analysis of variance was used to compare continuous variables among the groups. A 2-tailed Student’s *t* test or nonparametric Kruskal–Wallis, Mann–Whitney, or Wilcoxon’s signed rank tests were used to assess statistical significance. A Tukey correction was performed to adjust for multiple to one comparison. *p* < 0.05 was considered to indicate statistical significance.

## 3. Results

### 3.1. Evogliptin Suppresses Calcified Lesion Formation in eNOS^-/-^ Mice

**Figure 1:**
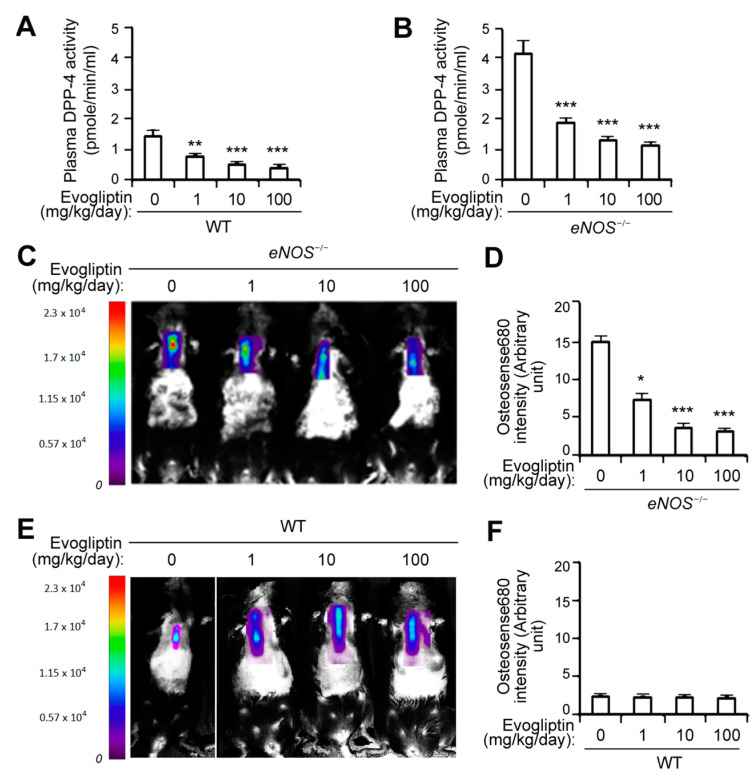
Attenuation of in vivo calcific lesion formation by evogliptin. (**A**,**B**) Plasma DPP-4 activity was measured after 12 weeks of evogliptin administration (1, 10, and 100 mg/kg/day) in wild type (WT) (**A**) and *eNOS*^−/−^ (**B**) mice (*n* = 6 per group). (**C**) *eNOS*^−/−^ mice were administered with vehicle or evogliptin (1, 10, or 100 mg/kg/day) orally for 12 weeks. Calcific lesion formation in vivo was determined using molecular imaging with Optix MX3 after the injection of a fluorescent agent (Osteosense680). (**D**) Quantification of fluorescence in *eNOS*^−/−^ mice (*n* = 6 per group). (**E**) Wild type (WT) mice administrated with evogliptin were injected with Osteosense680 and calcification was determined. (**F**) Quantification of fluorescence in WT mice (*n* = 6 per group). Values represent the fold changes of the mean value measured following vehicle treatment. * *p* < 0.05, ** *p* < 0.005, *** *p* < 0.0005 versus vehicle control. Data represent the mean ± SD. *p* values were obtained using a Kruskal–Wallis test.

We evaluated the efficacy of evogliptin for preventing calcific lesion formation in *eNOS*^−/−^ mice, which is an animal model of CAVD [25,26], via the molecular imaging of calcification in vivo after the injection of a fluorescent bisphosphonate-conjugated molecular agent (Osteosense680). As expected, evogliptin administration led to significant reduction in plasma DPP-4 activity in both WT (Figure 1A) and *eNOS*^−/−^ mice (Figure 1B), with an elevated DPP-4 protein level in plasma [19]. Fluorescence reflectance imaging revealed that the administration of evogliptin potently decreased the in vivo calcification of *eNOS*^−/−^ mice at a 1 mg/kg/day dosage, and further reduced calcification at 10 and 100 mg/kg/day levels, compared with the control animals receiving vehicle treatment (Figure 1C,D). In wild-type (WT) mice, however, a much lower level of calcification was evident and evogliptin had no significant effect on calcific nodule formation (Figure 1E,F). These results indicated that evogliptin suppresses calcific deposition in an in vivo model of CAVD in the mouse.

### 3.2. Evogliptin Attenuates the Osteogenic Transition of Aortic Valvular Interstitial Cells in Association with a Reduced Expression of Osteogenesis-Related and Fibrosis-Associated Genes

**Figure 2:**
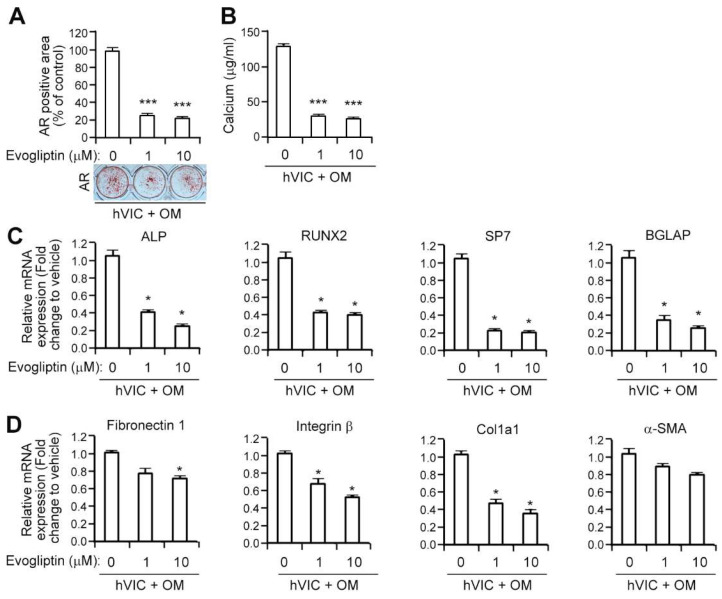
Evogliptin attenuates the osteogenic transition of aortic valvular interstitial cells by reducing the transcript expression of osteogenesis-associated genes in vitro. (**A**) After 3 weeks of osteogenic stimulation of human valvular interstitial cells (hVICs) in the presence or absence of evogliptin (1 or 10 μM), alizarin red (AR) staining was conducted (lower). The bar graph presents the relative alizarin red-positive area measured in each culture dish (upper). (**B**) The calcium concentration in the cells shown in (**A**) was measured. (**C**) Quantitative reverse transcription polymerase chain reaction (qPCR) analysis was performed to evaluate mRNA expression levels of ALP (alkaline phosphatase), RUNX2 (Runt-related transcription factor 2), Sp7 (Sp7 Transcription Factor), and BGLAP (bone gamma-carboxyglutamic acid-containing protein) in hVICs in the presence of the indicated concentrations of evogliptin. (**D**) The transcript levels of fibrosis-related genes including fibronectin 1, integrin β, Col1a1, and α-smooth muscle actin (α-SMA) were determined using qPCR in hVICs in the presence of various concentrations of evogliptin. * *p* < 0.05, *** *p* < 0.0005 versus vehicle control. Data represent the mean ± SEM. *p* values were obtained using a Kruskal–Wallis test.

Given the inhibitory effects of evogliptin on calcific nodule formation in vivo, we next examined whether this agent could inhibit the osteogenic changes of human valvular interstitial cells (hVICs) in vitro. We isolated VICs from human aortic valve tissues and induced their osteogenic differentiation with osteogenic media. Evogliptin treatment of these cells led to significant reduction (about 80%) in matrix mineralization, as revealed by alizarin red staining, at a concentration as low as 1 μM compared to vehicle, and this stayed reduced at a 10 μM evogliptin dose (Figure 2A). Notably, the reduction in alizarin red staining was associated with a parallel decrease in the calcium concentrations in VICs (Figure 2B). Taken together, these results suggested that evogliptin can significantly attenuate the calcium deposition of hVICs in vitro.

Given that the upregulation of transcription factors is related to the osteogenic differentiation of hVICs when grown osteogenic media [19], we next examined whether the attenuation by evogliptin of the osteogenic conversion of VICs is involved in the reduction of osteogenesis-related gene expression. To this end, we evaluated the transcript levels of these genes using qPCR followed by osteogenic differentiation. Evogliptin treatment of VICs at a 1 μM dose reduced the mRNA expression of alkaline phosphatase (ALP), an enzyme crucially required for biomineralization, and this reduction was sustained at a 10 μM evogliptin concentration (Figure 2C). The transcript expression levels of other bone markers, including runt-related transcription factor 2 (RUNX2), Sp7 transcription factor (also called osterix), and bone γ-carboxyglutamic acid-containing protein (BGLAP, also known as osteocalcin) were also dramatically reduced by evogliptin (Figure 2C). Previous clinical observations have suggested that fibrosis is a common complication of aortic valve stenosis [33]. To evaluate the effects of evogliptin on transcript expression of fibrosis-related genes, we examined the mRNA expression of these genes in VICs following exposure to evogliptin and found lower mRNA levels for fibronectin 1, integrin β, and col1a1, but not α-SMA as determined by qPCR (Figure 2D), indicating that evogliptin may be associated with the inhibition of fibrosis.

### 3.3. Evogliptin Attenuates the Expression of Proinflammatory Cytokines

**Figure 3:**
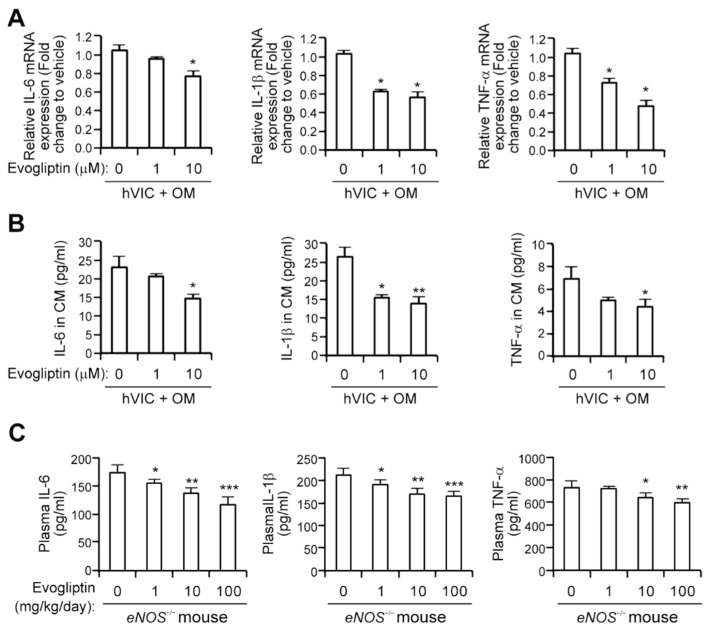
Evogliptin attenuates the expression of proinflammatory cytokines. (**A**) qPCR analysis was conducted to evaluate mRNA expression levels of IL-6, IL-1β, and TNF-α in hVICs in the presence of the indicated concentrations of evogliptin. (**B**) The protein levels of IL-6, IL-1β, and TNF-α in the conditioned media from hVICs treated with indicated concentrations of evogliptin were determined utilizing ELISA. (**C**) IL-6, IL-1β, and TNF-α plasma concentrations in the mice were determined using ELISA (*n* = 6, each group) at 12 weeks after the administration of evogliptin. * *p* < 0.05, ** *p* < 0.005, *** *p* < 0.0005 versus vehicle control. Data represent the mean ± SD. *p* values were obtained using a Kruskal-Wallis test.

Proinflammatory cytokines have been suggested to play a pivotal role in CAVD pathobiological progression [4,5,6]. We thus examined whether evogliptin reduces the expression of these cytokines using qPCR analysis and observed attenuated transcript levels of IL-6, IL-1β, and TNF-α in hVICs (Figure 3A). Moreover, the protein levels of these cytokines in the conditioned media of hVICs were significantly reduced by evogliptin (Figure 3B). Next, the concentrations of IL-6, IL-1β, and TNF-α protein in plasma from *eNOS*^−/−^ mice treated with vehicle or evogliptin (1, 10, or 100 mg/kg/day) for 12 weeks were measured using ELISA. Evogliptin administration downregulated the plasma level of IL-6 protein (Figure 3C). Likewise, the secreted IL-1β, and TNF-α protein levels in the plasma were also reduced by evogliptin treatment (Figure 3C), suggesting that evogliptin attenuates proinflammatory cytokine expression.

### 3.4. Evogliptin Attenuates Calcific Aortic Valve Stenosis in a Rabbit Model of CAVD

**Figure 4:**
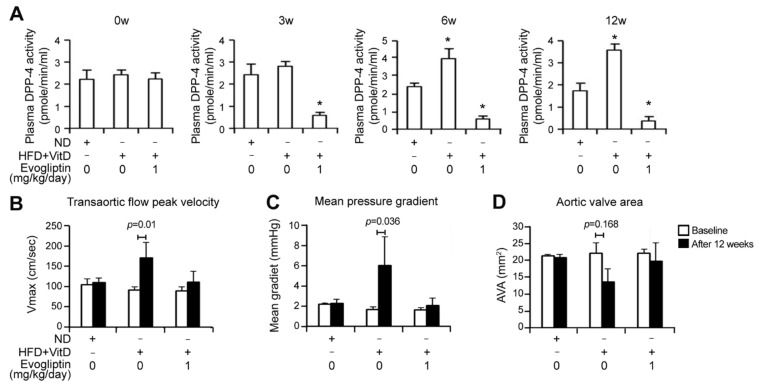
Effects of evogliptin on the aortic valve in a rabbit model of calcific aortic valve disease as determined by echocardiographic assessment. (**A**) Plasma DPP-4 activity was measured at baseline (0 weeks) and at 3, 6, or 12 weeks in the control group fed with a normal diet (ND), in rabbits fed a high-cholesterol diet and vitamin D2 supplements (HFD + VitD), and in evogliptin-treated rabbits on that same diet (HFD + VitD + evogliptin). Each group comprised ≥5 animals. (**B**,**C**) Aortic valve function was assessed by continuous-wave Doppler to measure the integral of the transvalvular flow and record the transaortic peak velocity (Vmax) (**B**) and mean pressure gradients (**C**) at baseline and at 12 weeks after treatment initiation in the control (ND), HFD + VitD, and HFD + VitD + evogliptin groups. Each group comprised ≥5 animals. (**D**) The standard continuity equation for echocardiography was used to measure the aortic valve area (AVA). Data represent the mean ± SD. *p* values were obtained using nonparametric matched data analysis and a Wilcoxon’s signed rank test.

To further evaluate the effects of evogliptin on the pathogenesis of aortic valve stenosis, we employed a CAVD rabbit model. A combination diet with high-cholesterol and vitamin D (VitD) supplements has been shown to induce aortic valve stenosis and calcium deposition in rabbits [34,35] and increase DPP-4 activity [36]. Indeed, we found in our present experiments that the HFD + VitD group showed an increase in the plasma DPP-4 activity by approximately 2-fold when compared with the control group at 6 and 12 weeks, further supporting the involvement of DPP-4 activity in the pathogenesis of aortic stenosis. On the other hand, such an enhancement in DPP-4 activity was significantly attenuated in the evogliptin-treated group over a 12-week period (Figure 4A), as expected, confirming the inhibition of DPP-4 activity by this drug.

An important finding of our current analysis was that the integral of the transvalvular flow measured using continuous-wave Doppler showed a significantly increased transaortic maximal velocity and mean transaortic pressure gradients in the HFD + VitD group at 12 weeks, whereas the evogliptin treated group showed no significant change in these Doppler parameters (Figure 4B,C). The HFD + VitD rabbit group displayed a modest increase in the aortic valve area (*p* = 0.168), as measured with a standard continuity equation, whilst evogliptin treatment reversed the aortic valve area to a similar level to that seen in the control rabbits (Figure 4D). These results indicated the beneficial effects of evogliptin through the attenuation of HFD + VitD-induced aortic valve stenosis in vivo.

**Figure 5:**
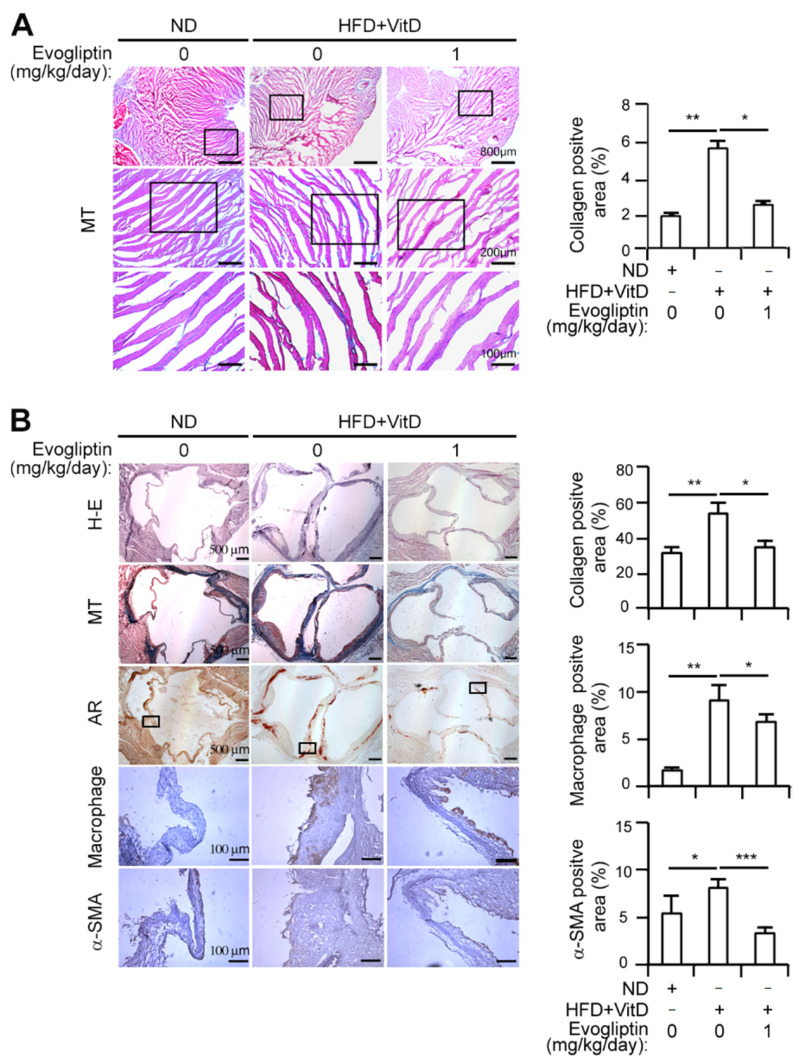
Effects of evogliptin on the aortic valve in a rabbit model with calcific aortic valve disease as determined by immunohistochemical staining. (**A**) Masson trichrome (MT) staining was performed to detect cellularity in the myocardium of a calcific aortic valve disease (CAVD) rabbit model showing extracellular matrix (ECM) accumulation (green–blue staining) (left). Quantitative analysis of the fibrotic area in the myocardium (right). (**B**) Representative photographs from light microscopy observations of the rabbit aortic valves with immunohistochemical staining for macrophage and α-SMA also shown (left). Hematoxylin and eosin (H–E), Masson trichrome (MT), and alizarin red (AR) staining were performed (left). Scale bar, 500 or 100 μm. The areas positive for collagen, macrophages, and SMA were quantitated, respectively (right). * *p* < 0.05, ** *p* < 0.005, *** *p* < 0.0005 versus normal diet (ND) control. Data represent the mean ± SD. *p* values were obtained using nonparametric matched data analysis and a Wilcoxon’s signed rank test.

The extent of myocardial fibrosis assessed by Masson trichrome staining showed an approximately 3-fold increase in myocardial fibrosis in the HFD + VitD group compared to the normal diet group, an increase which was abrogated by evogliptin treatment (Figure 5A). Furthermore, HFD + VitD-mediated calcium deposition was reduced by evogliptin administration, as assessed by alizarin red staining (Figure 5B). Hematoxylin-eosin staining revealed that the HFD + VitD rabbits showed a modestly increased thickening of the aortic valve leaflets, accompanied by an increase in α-SMA staining which is indicative of a myofibroblast phenotype [37], compared to control rabbits (Figure 5B). Conversely, the evogliptin-treated rabbits exhibited relatively thin cusps and less α-SMA staining than the HFD + VitD group (Figure 5B). With respect to inflammation, evogliptin reduced the HFD + VitD-mediated increase in macrophage infiltration in the aortic cusps of the rabbits (Figure 5B). Collectively, these results revealed beneficial effects of evogliptin on proinflammatory processes, against the progression of fibrosis, and against calcific nodule formation in vivo.

## 4. Discussion

An increasing amount of evidence now associates the inhibition of DPP-4 activity with positive effects on the incidence of cardiovascular events [38,39,40]. A *DPP-4*-deficient rat has shown better cardiac performance due to protection against ischemia/reperfusion injury [39]. DPP-4 deficiency and DPP-4 inhibition by sitagliptin has been found to reduce mouse mortality due to a lower rate of myocardial infarction [40]. We have previously shown from our laboratory that the DPP-4 inhibitor sitagliptin reduces the mineralization of VICs in vitro, decreases calcific lesion formation in *eNOS*^−/−^ mice, and improves aortic valve performance accompanied by a reduction in the calcium deposits in a rabbit CAVD model [19]. Moreover, we also reported that aortic stenosis patients who received DPP-4 inhibitors that have a higher cardiac tissue distribution profile show a significantly lower risk of severe aortic stenosis progression than patients who did not receive DPP-4 inhibitors, or were treated with those having a lower cardiac tissue distribution profile [20]. We also found that evogliptin had the highest cardiac tissue distribution among the eight known DPP-4 inhibitors (evogliptin, linagliptin, gemigliptin, alogliptin, sitagliptin, vildagliptin, saxagliptin, and teneligliptin) as revealed by pharmacokinetic and pharmacodynamic analysis [20]. In our present study, we have provided several lines of evidence that pharmacological inhibitor evogliptin, which shows the highest cardiac tissue distribution among its class of molecules, also has positive effects on the incidence of CAVD. Several clinical trials of evogliptin administration among healthy subjects without diabetes showed good tolerability within the dose range of 1.25–60 mg and dose-dependent DPP-4 inhibition: no subjects developed hypoglycemia [41,42]. In addition, long-term clinical outcome studies using different DPP-4 inhibitors revealed that development of adverse clinical events including heart failure and cardiovascular death did not increase with chronic DPP-4 inhibition [43,44,45].

The pathobiological progression of CAVD involves the activation of VICs, infiltration by inflammatory cells, fibrosis of the valve leaflets, and the formation of calcific lesions on the surface of the leaflets [4,5,6]. VICs are heterogeneous cells that are inducible to an osteogenic lineage and form calcified nodules in culture [46]. In our current study, the inhibition of DPP-4 activity by evogliptin led to a reduced osteogenic differentiation and mineralization of VICs (Figure 2), indicating the inhibitory effects of evogliptin on the osteogenic transition of VICs. It was interesting to note that evogliptin reduced the calcium deposition in vivo in both *eNOS*^−/−^ mice (Figure 1) and in a rabbit CAVD model (Figure 5). However, the clinical efficacy of evogliptin, including its distribution in organs and tissues, and its optimal dose for attenuating the non-glycemic function of DPP-4 to halt CAVD progression, remain to be fully elucidated.

Several previous lines of evidence have suggested that the inhibition of DPP-4 activity has potential as anti-inflammatory effects. DPP-4 inhibitors suppress cytokine production [14], inhibit monocyte infiltration to tissue niches [36], reduce the disease severity of rheumatoid arthritis in an animal model [16], suppress the inflammatory response in association with multiple sclerosis [47], and suppress the symptoms of inflammatory bowel disease [48]. Our current observations that evogliptin reduces the levels of TNF-α, IL-1β, and IL-6 proteins both in VICs and in the plasma of *eNOS*^−/−^ mice (Figure 3) and inhibits macrophage infiltration in a rabbit CAVD model (Figure 5), may also be relevant to the putative anti-inflammatory properties of DPP-4 inhibitors. Our present observations of an evogliptin-mediated reduction in proinflammatory cytokines (Figure 3) are compatible with the findings of a prior report that linagliptin significantly decreases the TNF-α, IL-1β, and IL-6 transcript levels in an animal model [14] and anagliptin suppresses macrophage infiltration and reduces the production of TNF-α and IL-6 [49] that promotes an osteogenic program and the mineralization of VICs [50]. Notably, however, our current analyses did not clarify whether evogliptin suppresses the function of lymphocytes directly or whether the substrates of DPP-4 are associated with the anti-inflammatory effects of evogliptin.

Recently, a link between inflammation and calcification has been suggested via regulating RUNX2 by nuclear factor kappa B (NF-κB) [51]. NF-κB is one of the main mediators of inflammation that plays a crucial role in the initiation and progression of CAVD [52]. RUNX2 is a key osteogenic transcription factor that initiates an osteoblastic differentiation and mineralization of VICs [53]. Nguyen et al. showed that evogliptin ameliorated the activation of NF-κB [54] and we found that evogliptin dramatically reduced RUNX2 expression (Figure 2C). Given the evogliptin-mediated inhibition of both NF-κB and RUNX2, it is possible that evogliptin may influence the interaction between inflammation and calcification via NF-κB/RUNX2, however, the precise role of evogliptin in this interaction is required to be elucidated.

Previous reports have suggested that the DPP-4 inhibitor linagliptin suppresses the profibrotic program by inhibiting the endothelial-to-mesenchymal transition [55] and that sitagliptin reduces myocardial fibrosis and the expression of TGF-β1 in the db/db mouse, which is an animal model of diabetes and obesity [18]. These results concur with our present observations that Col-1a1 and Fn-1, which are pro-fibrotic factors, are suppressed by evogliptin (Figure 2) and the reduction of myocardial fibrosis by evogliptin (Figure 5), indicating that this DPP-4 inhibitor may have pleiotropic anti-fibrosis effects. We could not reveal the detailed mechanisms or potential targets underlying the evogliptin-mediated attenuation of fibrosis, but suggest from our findings and other evidence that this drug suppresses tissue fibrosis. Further experiments are required to examine whether evogliptin reduces the expression of pro-fibrotic factors and extracellular matrix (ECM) deposition from fibroblasts and cardiomyocytes.

In summary, our current data suggest that evogliptin attenuates proinflammatory cytokine expression and fibrosis. Furthermore, we here provide evidence that evogliptin suppresses calcific nodule formation and improves aortic stenosis in animal models. These observations are consistent with the observed beneficial effects of DPP-4 inhibitors on cardiac health and provide insights into the usage of evogliptin, a known DPP-4 inhibitor, to protect against the development of calcific disease.

## Data Availability

The data presented in this study are available within this article.

## Abbreviations

AVA, aortic valve area; BGLAP, γ-carboxyglutamic acid-containing protein; CAVD, calcific aortic valve disease; Chol, high-cholesterol diet; DPP-4, dipeptidyl peptidase-4; ELISA, enzyme-linked immunosorbent assay; GAPDH, glyceraldehyde-3-phosphate dehydrogenase; H&E, hematoxylin and eosin; MT, Masson trichrome stain, ND, normal diet; qPCR, quantitative real-time polymerase chain reaction; RUNX2, runt-related transcription factor 2; TGF-β, transforming growth factor-β; VIC, valvular interstitial cell; vitD, vitamin D2; Vmax, peak velocity.
